# Role of cannabinoid receptor 1 in human adipose tissue for lipolysis regulation and insulin resistance

**DOI:** 10.1007/s12020-016-1172-6

**Published:** 2016-11-17

**Authors:** Cherno O. Sidibeh, Maria J. Pereira, Joey Lau Börjesson, Prasad G. Kamble, Stanko Skrtic, Petros Katsogiannos, Magnus Sundbom, Maria K. Svensson, Jan W. Eriksson

**Affiliations:** 1 Department of Medical Sciences, Uppsala University, Uppsala, Sweden; 2 AstraZeneca R&D, Mölndal, Sweden; 3 Department of Endocrinology, Institute of Medicine, Sahlgrenska Academy, University of Gothenburg, Gothenburg, Sweden; 4 Department of Surgical Sciences, Uppsala University, Uppsala, Sweden

**Keywords:** Type 2 diabetes, Glucocorticoids, Insulin resistance, Adipose tissue, Endocannabinoid system

## Abstract

We recently showed that the peripheral cannabinoid receptor type 1 (*CNR1*) gene is upregulated by the synthetic glucocorticoid dexamethasone. CNR1 is highly expressed in the central nervous system and has been a drug target for the treatment of obesity. Here we explore the role of peripheral CNR1 in states of insulin resistance in human adipose tissue. Subcutaneous adipose tissue was obtained from well-controlled type 2 diabetes subjects and controls. Subcutaneous adipose tissue gene expression levels of *CNR1* and endocannabinoid synthesizing and degrading enzymes were assessed. Furthermore, paired human subcutaneous adipose tissue and omental adipose tissue from non-diabetic volunteers undergoing kidney donation or bariatric surgery, was incubated with or without dexamethasone. Subcutaneous adipose tissue obtained from volunteers through needle biopsy was incubated with or without dexamethasone and in the presence or absence of the CNR1-specific antagonist AM281. *CNR1* gene and protein expression, lipolysis and glucose uptake were evaluated. Subcutaneous adipose tissue *CNR1* gene expression levels were 2-fold elevated in type 2 diabetes subjects compared with control subjects. Additionally, gene expression levels of *CNR1 *and endocannabinoid-regulating enzymes from both groups correlated with markers of insulin resistance. Dexamethasone increased *CNR1* expression dose-dependently in subcutaneous adipose tissue and omental adipose tissue by up to 25-fold. Dexamethasone pre-treatment of subcutaneous adipose tissue increased lipolysis rate and reduced glucose uptake. Co-incubation with the CNR1 antagonist AM281 prevented the stimulatory effect on lipolysis, but had no effect on glucose uptake. CNR1 is upregulated in states of type 2 diabetes and insulin resistance. Furthermore, CNR1 is involved in glucocorticoid-regulated lipolysis. Peripheral CNR1 could be an interesting drug target in type 2 diabetes and dyslipidemia.

## Introduction

Obesity and type 2 diabetes (T2D) are recognized as major health problems of epidemic proportions worldwide. Obesity, in particular central obesity, increases the risk of cardiovascular disease, insulin resistance and T2D. It is estimated that globally more than 1.9 billion adults are overweight and 9 % of adults are diabetic [1]. From a public health perspective it is of interest to identify and explore mechanisms and potential treatment concepts that are common for insulin resistance and obesity because of their shared association with the onset of T2D.

Glucocorticoids are steroid hormones whose synthetic analogs are used clinically for the treatment of autoimmune or inflammatory conditions [2]. Due to their immunosuppressive properties they are also used in transplant patients to prevent graft rejection. However, elevated plasma glucocorticoid levels, such as in Cushing’s syndrome or during long-term treatment, are associated with several adverse effects such as obesity, dyslipidemia, insulin resistance, and the onset of T2D [3]. The identification of glucocorticoid-regulated genes that are associated with insulin resistance or obesity can provide novel pharmacological approaches for such conditions. In a previous microarray study [4], using a model of insulin resistance by incubating human adipose tissue with the synthetic glucocorticoid dexamethasone, cannabinoid receptor type 1 (*CNR1)* was identified as one of the genes with the greatest increase in expression in subcutaneous and omental adipose tissue (SAT and OAT, respectively). CNR1 is a member of the cannabinoid receptor family and the superfamily of G protein-coupled receptors recognized to activate multiple signaling pathways regulating cell survival/death and energy metabolism [5]. The highest expression levels of *CNR1* are observed in different brain regions, but it is also present at lower levels in most other cells/tissue types, including adipose tissue [6, 7].

The endocannabinoid system, composed of CNR1 and CNR2, their lipid ligands (endocannabinoids) 2-arachidonoylglycerol (2-AG) and anandamide (AEA), and the endocannabinoid synthesis and degrading enzymes; plays an important role in the regulation of energy homeostasis [8, 9]. 2-AG is synthesized by diacylglycerol lipase (DAGL) and degraded by monoacylglycerollipase (MGL). While AEA is synthesized by *N*-acyl phosphatidylethanolamine phospholipase D (NAPE-PLD) and degraded by fatty acid amide hydrolase (FAAH) [10]. DAGL enzymes are encoded by two separate genes, denoted *DAGL-ALPHA* and *DAGL-BETA*.

An association between glucocorticoids and the endocannabinoid system has previously been demonstrated; where glucocorticoids elevate expression of endocannabinoids in regulation of the hypothalamic-pituary-adrenal axis [9, 11]. CNR1 regulates food intake in the hypothalamus [12] and in obesity the endocannabinoid/CNR1 system is upregulated, both centrally and peripherally [13, 14]. Given the role of CNR1 in obesity, antagonists have been developed as anti-obesity drugs. In 2006, a potent and selective CNR1-antagonist, rimonabant, was approved for treatment of obesity and for overweight patients with metabolic comorbidities such as T2D [15]. However, due to reported side effects such as depression and anxiety, rimonabant was withdrawn from the market [16]. Although the association between the central and peripheral levels of CNR1 and obesity has been demonstrated, it is uncertain if an increase of CNR1 in adipose tissue is sufficient to induce changes in glucose and lipid metabolism. Prior studies have attempted to separate the brain effects of endocannabinoids from their peripheral effects [17, 18]. However, these studies have been inconclusive since they have lacked a peripherally restricted CNR1 antagonist.

In this study we aim to investigate if CNR1 is a factor associated with the development of insulin resistance in adipose tissue by the examination of the endocannabinoid system in freshly harvested SAT from healthy control vs. T2D subjects. In addition, we aim to, via glucocorticoid-induced insulin resistance by long-term incubation (24 h) of SAT; investigate whether CNR1 plays a role in the regulation of glucose and lipid metabolism in human adipocytes.

## Materials and methods

### Adipose tissue donors

A cohort of 20 T2D subjects was group-wise matched with 20 non-diabetic subjects by gender (10F/10M), age (58 ± 9 vs. 58 ± 11) and body mass index (BMI) (30.7 ± 4.9 vs. 30.8 ± 4.6 kg/m^2^) [19]. Fasting blood samples, and oral glucose tolerance test (OGTT) and SAT needle biopsies were performed as previously described [19]. SAT was acquired by needle aspiration of the lower abdominal region and used to assess the endocannabinoid system and measure adipocyte glucose uptake [19]. Clinical and biochemical characteristics of the subjects are shown in Supplementary Table 1. A schematic view of the study is given in Supplementary Fig. 1A.

In a separate cohort, paired samples of human SAT and OAT were obtained from non-diabetic subjects with a wide distribution of BMI and insulin sensitivity (13M/31F, 24–66 years, BMI 20.7–56.3 kg/m^2^) undergoing kidney donation (*n* = 35) at the Sahlgrenska University Hospital or bariatric surgery (*n* = 9) at the Uppsala University Hospital. Paired SAT and OAT were used to study the *CNR1* mRNA expression levels (*n* = 41) and the effects of dexamethasone on *CNR1* mRNA (*n* = 30) and protein expression (*n* = 5) and glucose uptake (*n* = 12–21). In addition, SAT was obtained from a separate group of non-diabetic volunteers (5M/21F, 21–72 years, BMI 21.3–32.9 kg/m^2^) by needle aspiration of the abdomen after local dermal anesthesia with lidocaine (Xylocain; AstraZeneca, Sweden). These adipose tissue samples were used to study the effects of dexamethasone treatment and a CNR1-antagonist or CNR1-agonist on adipocyte lipolysis (*n* = 19) and glucose uptake (*n* = 12). Due to limited amounts of adipose tissue obtained from biopsies, not all experiments were performed on samples from every subject. A representative schematic view of this part of the study is given in Supplementary Fig. 1B.

Fasting blood samples were collected for analysis of plasma glucose, insulin and lipids at the Department of Clinical Chemistry at the respective hospitals. Subjects with type 1 diabetes and/or T2D, other endocrine disorders, cancer or other major illnesses, as well as ongoing medication with beta-adrenergic blockers, systemic glucocorticoids or immune-modulating therapies were excluded from the study. Eighteen individuals were positive for having first-degree relatives with T2D. Among the 52 female subjects, 24 were pre-menopausal. Clinical and biochemical characteristics of the subjects are shown in Supplementary Table 2. Most of the subjects included in the lipolysis experiments were females (Supplementary Table 3).

The study protocols were approved by the Regional Ethics Review Boards in Gothenburg (Dnr 336-07) and Uppsala (Dnr 2013/330 and Dnr 2013-183/494). Written informed consent was obtained from all study participants.

### Endocannabinoid system in freshly harvested SAT

Immediately after the biopsies, the SAT from T2D and control subjects was snap frozen in liquid nitrogen. The gene expression levels of *CNR1* and the major enzymes responsible for the synthesis and degradation of the two principal endocannabinoids, 2-AG and AEA, was measured. 2-AG levels in SAT were also assessed but AEA levels were not detectable. Gene expression levels were obtained with RNA-Seq at Exiqon A/S, Vedback, Denmark and 2-AG quantification was done by Metabolon Inc’s (Durham, North Carolina, USA) TrueVision^TM^ as previously described [19].

### Adipose tissue incubation and assessments

Paired samples of SAT and OAT were cut into small pieces and incubated in DMEM containing 6 mM glucose (Invitrogen Corporation, Paisley, UK), 10 % FBS (Invitrogen) and 1 % PEST (Invitrogen) with or without the addition of dexamethasone (Sigma-Aldrich, St. Louise, MO, USA) at varying concentrations (0.003–3 µM), to assess the dose-response, or at a single optimal concentration (0.3 µM) for 24 h in 37 °C, 5 % CO_2_. Following incubation, part of the adipose tissue was snap-frozen for *CNR1* gene (real-time PCR) or protein (immunohistochemistry) expression analysis. Other parts of the incubated adipose tissue were used to isolate adipocytes with collagenase (Sigma), as previously described [20, 21], and glucose uptake was assessed in isolated adipocytes.

In addition, SAT was incubated in DMEM (6 mM glucose, 10 % FBS, 1 % PEST) with or without the glucocorticoid cortisol or dexamethasone (1 µM for both) and *CNR1* gene expression was measured. The potency of dexamethasone is ~5 times higher than cortisol, assessed as effects on *β*-adrenergic receptor expression (EC50 4.8 nmol/L for dexamethasone 24 nmol/L for cortisol) [22], and we have internal data showing a similar potency difference (not shown). Thus, 0.3 µM concentration of dexamethasone would correspond to a maximum physiological level of cortisol under stress conditions of about 1–2 µM [23]. To ensure a maximal effect on *CNR1* expression and compare the effects of dexamethasone and its natural glucocorticoid cortisol in *CNR1* mRNA expression, 1 µM was used. Moreover, SAT was incubated in DMEM (6 mM glucose, 10 % FBS, 1 % PEST) with or without dexamethasone (0.3 µM) for 24 h in 37 °C, 5 % CO_2_ and with or without the CNR1 antagonist/inverse agonist AM281 (1-(2,4-dichlorophenyl)-5-(4-iodophenyl)-4-methyl-*N*-4-morpholinyl-1*H*-pyrazole-3-carboxamide, Sigma, 3 µM) for the final 4 h of incubation. SAT was also incubated with or without the CNR1 agonist ACEA (Arachidonyl-2′-chloroethylamide, Cayman, 1 µM) for 24 h. Adipose tissue was used to test the effects of dexamethasone and the CNR1 antagonists on the adipocyte lipolysis and glucose uptake. In an acute setting, isolated fresh adipocytes were pre-incubated with the CNR1 antagonist AM281 (3 µM) for 30 min, which was then present during lipolysis. Lipolysis and glucose uptake were performed as previously described [20, 24].

Total RNA was isolated from adipose tissue and the RNA concentration was determined. RNA was then converted to cDNA and relative quantification of *CNR1* mRNA was performed. Frozen sections of adipose tissue incubated with or without dexamethasone were stained for CNR1 protein using immunohistochemistry.

Mitogen-activated protein kinase (MAPK) and lipolysis signaling was assessed by measuring protein levels and activation of extracellular signal-regulated kinase (ERK) and the key lipolytic protein hormone-sensitive lipase (HSL) in lysates of adipose tissue treated with or without dexamethasone and the CNR1 selective antagonist AM281 by immunoblotting. Immunoblotting was performed with equal amount of protein for all samples (10 µg) and with the use of primary antibodies to ERK (4695S, Cell Signaling Technology (CST), Danvers, MA, USA; diluted 1:1000) phospho-ERK (Thr202/Tyr204) (4370S, CST; diluted 1:1000), HSL (4107S, CST; diluted 1:1000) and phospho-HSL (Ser563) (4139S, CST; diluted 1:1000). GAPDH (5174S, CST; diluted 1:1000) was used as a loading control for all samples.

See Supplementary Materials and Methods for details.

### Statistical analysis

All data are presented as mean ± SEM, unless stated otherwise. All statistical analyses were performed using IBM SPSS Statistics software. The Kruskal-Wallis *H* Test was used to study differences in the *CNR1* gene expression in the dexamethasone dose-response. For comparison of *CNR1* gene expression between males and females in both SAT and OAT, data was log-transformed and one-way analysis of variance with Tukey’s Multiple Comparison post-hoc test was used. Differences between treatments in gene expression, glucose uptake and lipolysis for paired samples were determined using Wilcoxon signed-rank test, while Mann-Whitney U test was used to compare differences in gene expression between independent groups. Spearman’s bivariate correlation test was used to assess correlations between *CNR1* gene expression and metabolic parameters. Significant variables in the bivariate correlation analyses were subsequently included in multivariate stepwise regression analyses. A *p*-value < 0.05 was considered statistically significant.

## Results

### *CNR1* gene expression in freshly harvested SAT is elevated in T2D subjects and associated with markers of insulin resistance

**Fig. 1:**
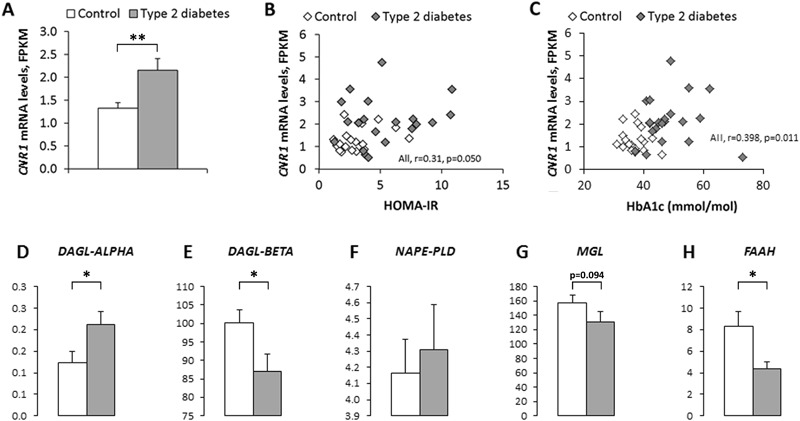
*CNR1* gene expression **a** is elevated in type 2 diabetes subjects (*n* = 20) compared with control subjects (*n* = 20). Bivariate correlation between *CNR1* mRNA expression levels in freshly harvested subcutaneous adipose tissue and HOMA-IR **b** and HbA1c **c**. Endocannabinoid-synthesizing **d–f** and degrading **g–h** enzymes are differentially expressed in SAT from type 2 diabetes subjects vs. controls. Mann-Whitney U test used to compare the differences between independent groups. * *p* < *0.05.* SAT was freshly harvested. *DAGL*, Diacylglycerol lipase; *NAPE-PLD*, *N*-acyl phosphatidylethanolamine phospholipase D; *MGL*, Monoacylglycerol lipase; *FAAH*, Fatty acid amide hydrolase; *CNR1*, Cannabinoid receptor type 1

Freshly harvested SAT from T2D subjects was found to have 1.6-fold higher (*p* < 0.01) *CNR1* gene expression levels compared with control subjects (Fig. 1a).

In bivariate correlation analyses, *CNR1* gene expression positively correlated with HbA_1c_, fasting glucose, 2 h glucose and glucose area under the curve (AUC) during OGTT and a trend of positive correlation with insulin and HOMA-IR (Table 1). There was no correlation with BMI or basal or insulin-stimulated adipocyte glucose uptake. After inclusion of glucose AUC during OGTT and HbA_1c_ in multivariate analyses, glucose AUC during OGTT (standard β coefficient = 0.44, *p* < 0.01; model: *r* ^2^ = 0.17) remained the only significant predictor of *CNR1* gene expression in SAT.

**Table 1:** Association between *CNR1*, *MGL*, *and FAAH* gene expression in freshly harvested SAT from healthy control subjects (*n* = 20) and T2D subjects (*n* = 20) and metabolic parameters

	*CNR1* mRNA		*MGL* mRNA		*FAAH* mRNA	
	*r*	*p*	*r*	*p*	*r*	*p*
HbA_1c_	**0.398**	**0.011**	**−0.387**	**0.014**	**−0.397**	**0.011**
HOMA-IR	0.310	0.052	**−**0.296	0.063	**−**0.262	0.103
Fasting insulin	0.301	0.059	**−**0.141	0.384	**−**0.191	0.237
Insulin AUC during OGTT	0.044	0.786	0.083	0.609	0.093	0.569
2 h Insulin during OGTT	0.069	0.673	**−**0.009	0.958	**−**0.037	0.821
Fasting glucose	**0.411**	**0.008**	**−0.506**	**0.001**	**−0.517**	**0.001**
Glucose AUC during OGTT	**0.375**	**0.017**	**−0.446**	**0.004**	**−0.430**	**0.006**
2 h Glucose during OGTT	**0.353**	**0.025**	**−0.394**	**0.012**	**−0.385**	**0.014**
HDL-cholesterol	−0.310	0.052	**0.427**	**0.006**	0.139	0.393
Triglycerides	0.193	0.232	−0.130	0.422	−0.191	0.238
BMI	0.017	0.919	0.111	0.497	0.159	0.329
Waist circumference	**−**0.148	0.362	**−**0.078	0.632	0.081	0.621
WHR	**−**0.004	0.978	**−0.436**	**0.005**	**−**0.217	0.179
SC adipocyte diameter	0.069	0.671	0.177	0.275	0.181	0.263
Basal glucose uptake	**−**0.093	0.584	**0.336**	**0.036**	0.245	0.134
25 µU insulin-stimulated glucose uptake	**−**0.095	0.575	**0.384**	**0.019**	0.299	0.073
1000 µU insulin-stimulated glucose uptake	**−**0.126	0.446	**0.385**	**0.015**	**0.367**	**0.022**

Bold values represent significant correlations as indicated by the *p*-values

Variables with *p*-value < 0.05 and gender were considered to multivariate stepwise regression analysis

*HbA*_*1c*_ glycosylated hemoglobin, *HOMA-IR* homeostatic model assessment of insulin resistance index, Glucose *AUC* Area under the glucose curve, *HDL-cholesterol* high-density lipoprotein cholesterol, *BMI* body mass index, *WHR* waist-hip ratior-values are Spearman correlation coefficients.

### 2-AG and endocannabinoid-regulating enzymes are associated with markers of insulin resistance

The gene corresponding to the endocannabinoid synthesizing enzyme *DAGL-ALPHA* was upregulated in T2D subjects compared with control subjects (*p* < 0.05, Fig. 1d), while *DAGL-BETA* was downregulated (*p* < 0.05, Fig. 1e). The expression levels of *NAPE-PLD* did not differ between the groups (Fig. 1f). The gene expression levels of the 2-AG-degrading enzyme *MGL* had a trend to be reduced in T2D subjects compared with control subjects (*p* = 0.094, Fig. 1g) while the gene expression levels of the AEA-degrading enzyme, *FAAH*, were lower in T2D subjects compared with control subjects (*p* < 0.05) (Fig. 1h).

The SAT levels of 2-AG did not differ between T2D subjects and control subjects (data not shown), but its levels negatively correlated with adipocyte basal and insulin-stimulated glucose uptake (*p* < 0.01, Supplementary Fig. 2A).

*MGL* positively correlated with basal and insulin-stimulated adipocyte glucose uptake (*p* < 0.05), HDL-cholesterol (*p* < 0.01) and negatively with HbA_1﻿c_, 2 h glucose during OGTT (*p* < 0.05), fasting glucose, glucose AUC during OGTT and waist-hip ratio (*p* < 0.01) (Table 1, Supplementary Fig. 2B). Following multivariate analysis where HbA_1c_, glucose AUC during OGTT, HDL and waist-hip ratio and basal and insulin-stimulated glucose uptake were included; HbA_1c_ (standard *β* coefficient = −0.36, *p* < 0.05) and HDL (standard *β* coefficient = 0.41, *p* < 0.01) remained significant predictors of *MGL* expression (model: *r* ^2^ = 0.32, *p* < 0.001).

*FAAH*, positively correlated with insulin-stimulated adipocyte glucose uptake (*p* < 0.05) and negatively correlated with HbA_1c_, ﻿2 h glucose during OGTT (*p* < 0.05), fasting glucose and glucose AUC during OGTT (*p* < 0.01) (Table 1, Supplementary Fig. 2C). Following multivariate analysis with 1000 µU insulin-stimulated glucose uptake, HbA_1c_, and glucose AUC during the OGTT; glucose AUC during the OGTT alone (standard β coefficient = −0.41, *p* < 0.01; model: *r* ^2^ = 0.15) remained a significant predictor of *FAAH*.

Neither of *DAGL-ALPHA* or *DAGL-BETA *or *NAPE-PLD* was found to correlate with markers of insulin resistance (data not shown).

### Glucocorticoids increase *CNR1* gene and protein expression in human adipose tissue

**Fig. 2:**
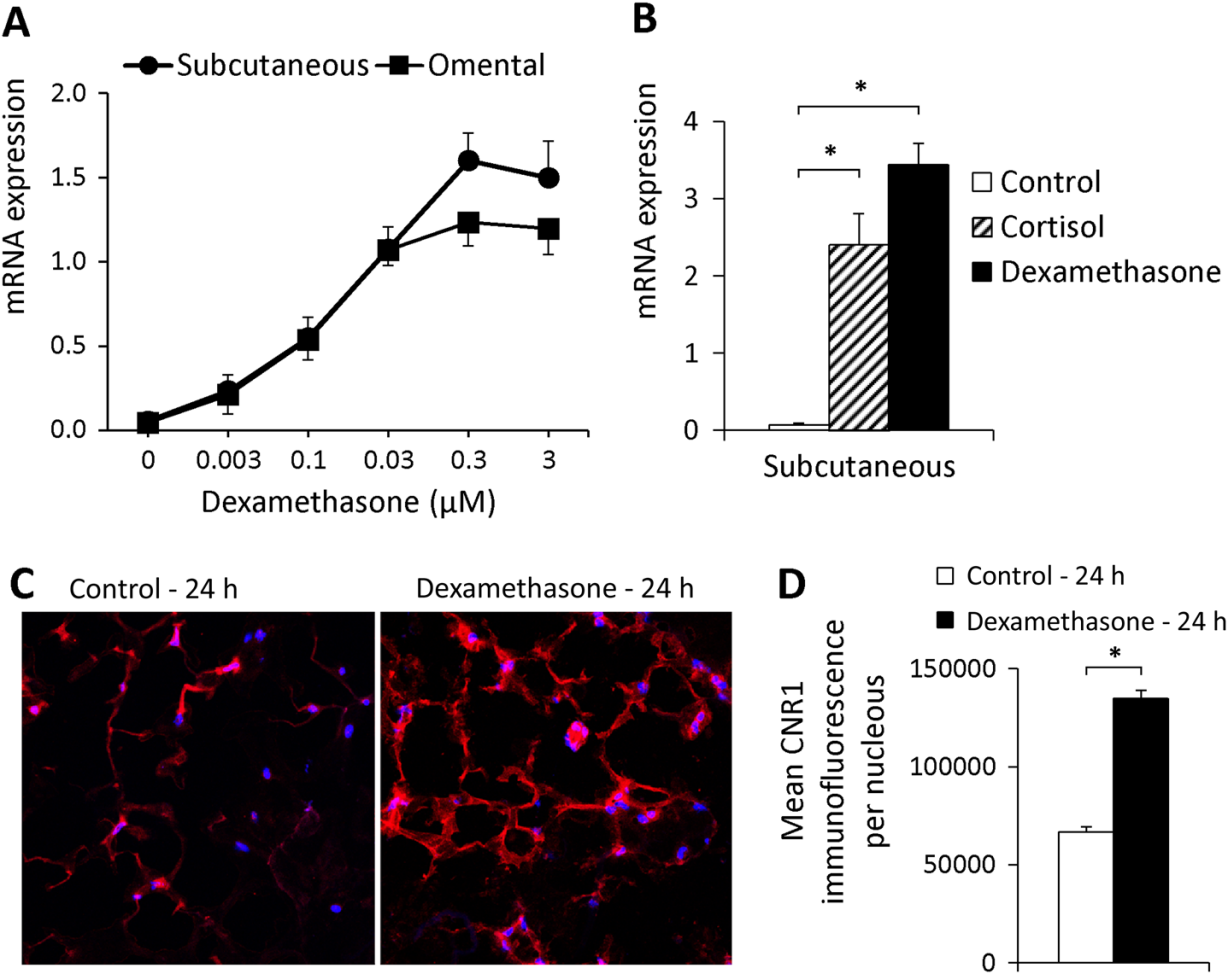
Glucocorticoids increase CNR1 gene and protein expression in adipose tissue. **a** 24 h incubation with dexamethasone (0.003–3 µM) increased the *CNR1* gene expression in a dose-dependent manner in SAT and OAT, compared with control (*n* = 3 for 0.003 µM; *n* = 9 for 0.1–0.03 µM; *n* = 10 for 0 and 0.3–3 µM), Kruskal-Wallis test, *** *p* < 0.001. **b** 24 h incubation with cortisol (1 µM) or dexamethasone (1 µM) increased *CNR1* gene expression to a similar level in SAT compared with control (*n* = 5), * *p* < 0.05. **c** Subcutaneous adipose samples incubated for 24 h with dexamethasone had a higher immunofluorescence staining of CNR1 (*red*) compared with non-treated samples (*n* = 5). Cell nuclear DNA was stained with DAPI (*blue*). A representative image is presented. **d** Average CNR1 immunofluorescence per nucleus of 5 independent experiments

The synthetic glucocorticoid dexamethasone (0.003–3 µM, *n* = 3–10) increased *CNR1* gene expression in a dose-dependent manner in both SAT and OAT during 24 h incubations by up to 25- and 22-fold, respectively (*p* < 0.001, Fig. 2a). The concentration-response curves show that a maximum effect on *CNR1* mRNA expression was exerted by 0.3 µM dexamethasone. A similar effect was exerted by the natural glucocorticoid cortisol (1 µM, *n* = 5, Fig. 2b). The glucocorticoid-induced upregulation was similar in adipose tissue from men and women. (Supplementary Fig. 4).

CNR1 protein expression in control and dexamethasone-treated SAT was assessed by immunohistochemistry. Dexamethasone treatment increased CNR1 protein amount by about 2-fold (*p* < 0.05, *n* = 5, Fig. 2c and d) compared with control (i.e., absence of dexamethasone).

See Supplementary Results for additional details.

### Associations between *CNR1* gene expression in long-term incubated adipose tissue and metabolic parameters

**Fig. 3:**
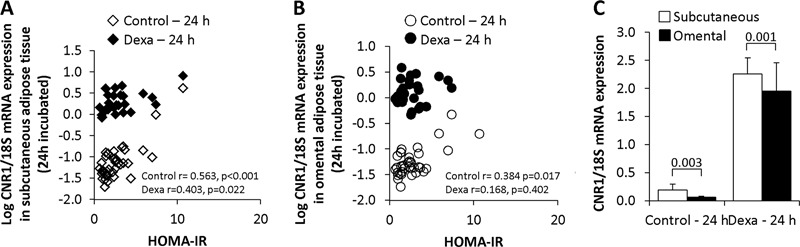
*CNR1* gene expression in 24 h incubated adipose tissue from the subcutaneous, but not the omental depot, is increased with insulin resistance. Bivariate correlation between *CNR1* mRNA expression in non-treated (control, *n* = 41) and dexamethasone-treated (*n* = 30) for 24 h paired samples of SAT **a** and OAT **b** and HOMA-IR. **c** *CNR1* mRNA expression levels in paired samples of SAT and OAT non-treated and dexamethasone-treated for 24 h (*n* = 41 and 30, respectively)

After adjustments in multivariate analyses following bivariate correlation analyses; HOMA-IR (standard *β* coefficient = 0.42, *p* < 0.001), waist circumference (standard *β* coefficient = 0.83, *p* < 0.001) and omental adipocyte diameter (standard *β* coefficient = −0.45, *p* < 0.001) remained significant predictors of *CNR1* gene expression in non-treated SAT (model: *r* ^2^ = 0.81, *p* < 0.001) (Table 2, Fig. 3 and Supplementary Fig. 3). In non-treated OAT only HOMA-IR (standard β coefficient = 0.65, *p* < 0.001) remained as a significant predictor of *CNR1* gene expression (model: *r* ^2^ = 0.43, *p* < 0.001) (Table 2, Fig. 3). Additionally, a negative correlation was found between the fold-change of SAT *CNR1* gene expression by dexamethasone, and HbA_1c_ (*r* = −0.47, *p* < 0.05) and a trend of negative correlation to HOMA-IR (*r* = −0.37, *p* = 0.061). In OAT, there was a trend of negative correlation between the *CNR1* fold change and HbA_1c_ (*r* = −0.35, *p* = 0.081). See Supplementary Results for additional details.

**Table 2:** Association between *CNR1* gene expression in 24 h non-treated and dexamethasone-treated SAT and OAT and metabolic parameters

	*CNR1* mRNA (24-h non-treated)								*CNR1* mRNA (24-h dexamethasone-treated)					
	SAT				OAT				SAT				OAT	
	Bivariate correlation^a^		Multivariate stepwise regression^b^		Bivariate correlation^a^		Multivariate stepwise regression^b^		Bivariate correlation^a^		Multivariate stepwise regression^b^		Bivariate correlation	
	*r*	*p*	Std *β*	*p*	*r*	*p*	Std *β*	*p*	*r*	*p*	Std *β*	*p*	*r*	*p*
Fasting insulin	**0.519**	**<0.001**	**–**	**–**	**0.402**	**0.012**	**–**	**–**	**0.381**	**0.031**	**–**	**–**	0.232	0.244
Fasting glucose	**0.322**	**0.029**			0.052	0.748	**–**	**–**	**0.419**	**0.012**	**–**	**–**	0.054	0.777
HbA_1c_	0.242	0.128	**–**	**–**	0.040	0.816	**–**	**–**	0.138	0.458	**–**	**–**	−0.035	0.865
HOMA-IR	**0.563**	**<0.001**	**0.415**	**<0.001**	**0.384**	**0.017**	**0.652**	**<0.001**	**0.403**	**0.022**		NS	0.168	0.402
HDL-cholesterol	**−0.470**	**0.001**		NS	**−0.387**	**0.014**		NS	**−0.214**	**0.024**		NS	−0.135	0.485
Triglycerides	0.251	0.097	**–**	**–**	0.233	0.148	**–**	**–**	0.002	0.989	**–**	**–**	0.040	0.835
BMI	**0.749**	**<0.001**		NS	**0.449**	**0.003**		NS	**0.420**	**0.012**		NS	0.168	0.375
Waist circumference	**0.623**	**<0.001**	**0.830**	**<0.001**	**0.441**	**0.004**		NS	**0.403**	**0.016**	**0.700**	**<0.001**	0.187	0.323
WHR	0.072	0.636	–	–	0.225	0.157	–	–	0.038	0.830	–	–	0.227	0.228
SC adipocyte diameter	**0.369**	**0.014**		NS	0.204	0.213	–	–	**0.370**	**0.029**		NS	0.133	0.482
OM adipocyte diameter	**0.507**	**<0.001**	**−0.453**	**<0.001**	0.169	0.303	–	–	**0.502**	**0.005**		NS	0.146	0.443

Bold values represent significant associations as indicated by the *p*-values

*SAT* subcutaneous adipose tissue, *OAT* omental adipose tissue, *Sc* subcutaneous, *Om* omental, *HbA*_*1c*_ glycosylated hemoglobin, *HOMA-IR* homeostatic model assessment of insulin resistance index, *HDL-cholesterol* high-density lipoprotein, *BMI* body mass index, *WHR* waist-hip ratio

^a^ r-values are Spearman correlation coefficients. Variables with *p*-value < 0.05 and gender were considered to multivariate stepwise regression analysis

^b^ Only the variables that had a *p*-value < 0.05 were considered in the final fitted model. Gender did not improve the prediction of the model. Std *β* are standard beta coefficients

### Effects of dexamethasone, AM281 and ACEA on lipolysis

**Fig. 4:**
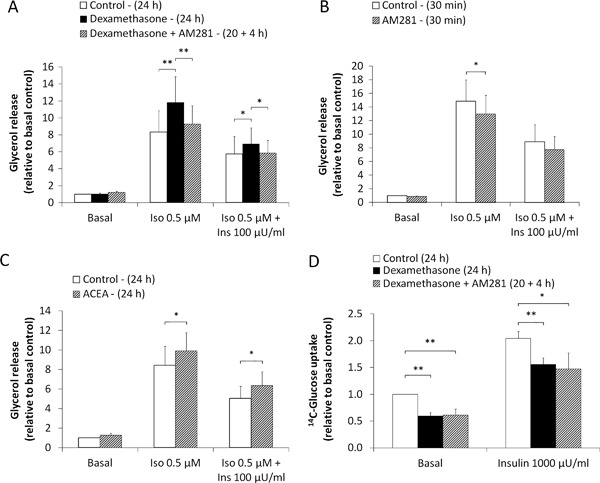
Effects of CNR1 antagonist/agonist on lipolysis and glucose uptake. **a** Effects of long-term incubation of SAT without (control) or with dexamethasone (0.3 µM) for 24 h or dexamethasone for 24 h plus the CNR1-specific antagonist AM281 (3 µM) for the final 4 h of incubation in basal, isoproterenol (0.5 µM) and isoproterenol plus insulin (100 µU/mL) adipocyte lipolysis (*n* = 10). **b** Effects of short-term incubation (30 min) of isolated adipocytes without (control) or with the CNR1-specific antagonist AM281 (3 µM) in basal, isoproterenol (0.5 µM) and isoproterenol plus insulin (100 µU/mL) adipocyte lipolysis (*n* = 5). **c** Effects of long-term incubation of SAT without (control) or the CNR1-specific agonist ACEA (1 µM) for 24 h of incubation in basal, isoproterenol (0.5 µM) and isoproterenol plus insulin (100 µU/mL) adipocyte lipolysis (*n* = 8). **d** Effects of long-term incubation of SAT without (control) or with dexamethasone (0.3 µM) for 24 h or dexamethasone for 24 h plus the CNR1-specific antagonist AM281 (3 µM) for the final 4 h of incubation in basal and insulin-stimulated (1000 µU/mL) adipocyte glucose uptake (*n* = 12)

Incubation of SAT (*n* = 10) with dexamethasone for 24 h significantly increased isoproterenol-stimulated lipolysis in adipocytes by 42 % (*p* < 0.01), compared with control (Fig. 4a). Addition of the CNR1-specific antagonist AM281 (3 µM) to the incubation media for the last 4 h of incubation prevented the dexamethasone effect on isoproterenol-stimulated lipolysis (*p* < 0.01, Fig. 4a). In addition, dexamethasone reduced the anti-lipolytic effect of insulin, while addition of AM281 prevented this effect (*p* < 0.05, Fig. 4a). Dexamethasone treatment, or co-incubation of dexamethasone with AM281, had no effects on basal lipolysis. Results from individual lipolysis measurements are reported in Supplementary Table 4.

Freshly harvested subcutaneous adipocytes that were pre-treated with AM281 for 30 min with the compound present throughout the lipolysis experiment showed a reduced isoproterenol-stimulated lipolysis by 11 % compared with non-treated adipocytes (*n* = 5, *p* < 0.05, Fig. 4b and Supplementary Table 5 for individual results).

In contrast, incubation of SAT for 24 h with the CNR1-specific agonist ACEA increased adipocyte isoproterenol-stimulated lipolysis by 17 % and counteracted insulin’s antilipolytic effect (*n* = 8, *p* < 0.01, Fig. 4c). Individual lipolysis measurements are reported in Supplementary Table 6.

### Effects of dexamethasone and AM281 on glucose uptake

Incubation of SAT with dexamethasone for 24 h significantly reduced adipocyte basal and insulin-stimulated glucose uptake by 40 and 30 %, respectively (*n* = *12, p* < 0.01, Fig. 4d). Addition of the CNR1-specific antagonist AM281 did not prevent the inhibitory effects of dexamethasone on glucose uptake.

The difference in SAT and OAT *CNR1* gene expression induced by dexamethasone after 24 h incubation was found to correlate negatively with the insulin-stimulated glucose uptake in both subcutaneous and omental dexamethasone-treated adipocytes (*p* < 0.05, Supplementary Fig. 5).

### Effects of dexamethasone and AM281 on MAPK and lipolysis signaling

**Fig. 5:**
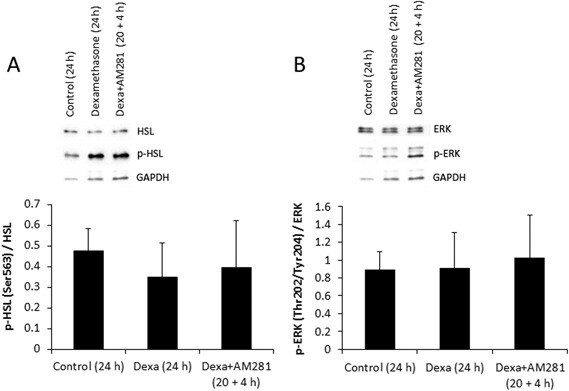
Incubation of adipose tissue with dexamethasone (0.3 µM) for 24 h or dexamethasone for 24 h together with the CNR1-specific antagonist AM281 (3 µM) for the final 4 h of incubation had no effect on the total protein levels or on the phosphorylation of HSL Ser563 **a** and ERK Thr202/Tyr204 **b** (*n* = 5). Data are means of densitometry analyses of p-HSL and p-ERK and normalized to the respective total protein levels (*n* = 5). GAPDH was used as a loading control protein

Incubation of SAT for 24 h with dexamethasone or co-incubation with dexamethasone and the CNR1 selective antagonist AM281 had no effects in total or phosphorylated levels of HSL or ERK (*n* = 5, Fig. 5a, b, respectively).

## Discussion

In this study we show that *CNR1* gene expression is elevated in states of insulin resistance and T2D. We also demonstrate that gene expression of endocannabinoid-degrading enzymes is reduced in T2D subjects and that this, together with elevated 2-AG levels, is associated with reduced glucose uptake capacity in adipocytes. This suggests a potential role of the peripheral endocannabinoid system to promote insulin resistance. However, whether CNR1 overexpression is a cause or a result of insulin resistance remains to be determined.

We also show that the synthetic glucocorticoid dexamethasone increases *CNR1* gene expression in human SAT and OAT in a dose-dependent manner. Our findings also imply that subjects with elevated insulin resistance have less elevation of *CNR1* gene expression by glucocorticoids compared with insulin sensitive subjects, possibly due to the already elevated levels of *CNR1* in insulin resistance states. The ability of dexamethasone to increase *CNR1* gene expression was expected, as we have previously shown in a microarray study that *CNR1* is one of the genes with the greatest increase in expression in human adipose tissue after dexamethasone treatment [4]. However, we could not demonstrate a correlation between gene and protein expression levels across the few individuals studied. The CNR1 protein levels were increased by about 2-fold in dexamethasone-treated SAT compared with control, while the mRNA levels were increased by about 25-fold. Although immunofluorescence is a powerful tool for determining the cellular distribution of an antigen, the extent of agreement between mRNA expression and semi-quantitative immunostaining data is usually poor [25]. Additionally, post-translational modifications of CNR1 have previously been reported [26]. Overall, the results suggest that incubation for 24 h with dexamethasone upregulates CNR1 mRNA and protein expression in adipose tissue.

There have been discordant results concerning *CNR1* expression in adipose tissue. We believe that experimental design is a possible contributor to the variability. We show that after 24 h incubation with no additional treatments, *CNR1* gene expression in both adipose tissue depots is positively correlated with several parameters of insulin resistance and central obesity (e.g., HOMA-IR, BMI, waist circumference and fat cell diameter). After adjustments in multivariate analyses, HOMA-IR, waist circumference and omental fat cell diameter remained significant predictors of subcutaneous *CNR1* gene expression while BMI was excluded. In OAT, only HOMA-IR remained a significant predictor of *CNR1* gene expression. Therefore, insulin resistance, rather than obesity, seems to be associated with *CNR1* gene expression in both SAT and OAT. Furthermore, our data in freshly harvested SAT showed that *CNR1* gene expression is increased in T2D subjects compared with controls, and associated with fasting glucose, glucose AUC during OGTT and HbA_1c_, but not with BMI. This discrepancy vs. the incubated tissue may suggest that culturing of adipose tissue per se affects *CNR1* gene expression. This is in agreement with a previous study showing no association between *CNR1* gene expression in adipose tissue and BMI in freshly harvested samples [27]. In contrast, increased *CNR1* expression with obesity has been shown in some reports [14, 28], but no multivariate corrections were performed and the number of subjects per group is limited (<10). In addition, others have found reduced *CNR1* expression in adipose tissue with obesity, but only post-menopausal women [6] or surgical patients with variation in age and concomitant medication were included [13]. In our study with freshly harvested samples we had well-controlled T2D subjects with a tight BMI and gender matching with control subjects, which allowed us to strictly compare the disease vs. the influence of obesity on *CNR1* gene expression.

Activity of the CNR1 depends on the endocannabinoid levels. Therefore, we measured the levels of one of the key endocannabinoids in the adipose tissue, 2-AG, and also expression levels of enzymes responsible for synthesis and degradation of 2-AG and AEA. 2-AG levels in adipose tissue did not differ between T2D and control subjects but were negatively associated with the adipocyte glucose uptake. In addition, we found that the gene expression levels of the endocannabinoids-degrading enzymes *FAAH* and *MGL* were reduced in T2D subjects compared with controls, and negatively associated with HbA_1c_. This is in agreement with several studies showing increased activity of the endocannabinoid system in T2D and/or obese subjects [29–31]. Altogether these findings suggest a potential role of the peripheral endocannabinoid-system in adipocyte metabolism and insulin resistance. One study measuring *FAAH* mRNA levels in SAT following hyperinsulinemic clamp showed a 2-fold elevation of *FAAH* mRNA in lean subjects that was not observed in the obese [32]. However, neither 2-AG levels or *FAAH* or *MGL* expression correlated with BMI in this study. We also found *DAGL-ALPHA* to be upregulated and *DAGL-BETA* downregulated in T2D subjects compared with controls. However, DAGL-ALPHA is reported to play a greater role than DAGL-BETA in 2-AG synthesis in adipose tissue [33]. It should be considered that other endocannabinoids and/or enzymes, as well as levels in other tissues and circulating levels are also of interest when exploring the activity of the endocannabinoid system.

We also explored the in vitro role of CNR1 in the glucocorticoid regulation of glucose and lipid metabolism in vitro in human subcutaneous adipocytes. To our knowledge, we show, for the first time, that a CNR1-specific antagonist, AM281, partly prevents the stimulatory effects of dexamethasone on lipolysis in adipocytes. A role of CNR1 in lipolysis is further supported by the effects of the CNR1-specific agonist, ACEA, to stimulate lipolysis. Elevated CNR1 expression levels may therefore be important for the regulation of lipolysis by glucocorticoids in human subcutaneous adipocytes and might contribute to the elevation of the FFA levels in circulation as observed in glucocorticoid-treated subjects [34, 35]. This might, in turn, contribute to ectopic fatty acid deposition in tissues, such as liver and skeletal muscle, and to insulin resistance and inflammation in these tissues [36]. Moreover, the observed inhibition of dexamethasone-induced lipolysis by AM281 mimics the insulin-mediated inhibition of lipolysis. The insulin suppression of isoproterenol-stimulated lipolysis by 30 % was modest. However, this suppression is similar to previous reports [19, 24] with identical in vitro incubation conditions. Moreover, we found that short-term treatment with the CNR1 antagonist, AM281, attenuated the lipolysis rate in freshly isolated adipocytes independent of dexamethasone-treatment. This suggests that CNR1 antagonism can regulate lipolysis independent of glucocorticoid-induced CNR1 expression levels, and also that AM281 may acutely affect lipolysis most likely by acting as an inverse agonist and reducing the intrinsic CNR1 activity. This indicates an involvement of CNR1 in the lipolysis regulation and suggests that peripherally restricted CNR1 antagonists via lowering of FFA levels may improve insulin sensitivity. In agreement with our findings, treatment of rats with CNR1 agonists stimulates lipolysis [37, 38], whereas a CNR1 antagonist [39] decreases plasma free fatty acids, supporting the notion of lipolysis being inhibited by CNR1-antagonism in vivo.

To explore possible mechanisms involved in the effects on the CNR1 activation of lipolysis we addressed the effects of dexamethasone and AM281 incubation in phosphorylation and protein levels of ERK1/2 and HSL in adipose tissue. ERK1/2 is a protein involved in the MAPK-pathway known to be regulated by the endocannabinoid system [40]. HSL is a key factor involved in lipolysis regulation by beta-adrenergic and insulin signaling, and is regulated by ERK [41, 42]. However, ERK and HSL activation and protein levels were not affected by dexamethasone or AM281 incubation. Future studies should thoroughly elucidate underlying mediators of lipolysis and their putative involvement in the action of CNR1 and its antagonists, as well as their interaction with beta-adrenergic and insulin signaling. Indeed, CNR1 has been shown to mediate glucocorticoid effects on AMPK activity in the hypothalamus of mice [43]. AMPK, being a mediator of lipolysis, is therefore another target of interest within the context of our study.

The inhibitory effect of dexamethasone on adipocyte glucose uptake is well known [44–46]. Our in vivo data also suggest an association between adipocyte glucose uptake and the levels of the endocannabinoid 2-AG and the gene expression of endocannabinoid-degrading enzymes *MGL* and *FAAH* in the adipose tissue. However, CNR1-specific antagonist/inverse agonist with AM281 did not affect dexamethasone inhibitory effect on glucose uptake in vitro. In contrast to our findings, CNR1 antagonism has previously been shown to improve tissue-specific glucose uptake in skeletal muscle [17] and in nucleus accumbens [47] from rats. In contrast, other in vitro studies indicated that glucose uptake is increased in murine 3T3-L1 adipocytes and human adipocytes by CNR1 agonism rather than antagonism [14, 48]. The discrepancies might be explained by different biological effects of the CNR1 antagonists/agonists in the different cell models used in these studies, e.g., rat skeletal muscle or brain tissue, murine cell lines or human adipocytes and in vivo or in vitro studies. In addition, different CNR1-specific compounds were used, e.g. the antagonists SR141716 or O-2050 [17, 47], the agonist WIN 55,212 [14, 47] or the endocannabinoids 2-AG or AEA [47, 48].

There are limitations to this study. This is primarily an in vitro study that does not take into consideration the complex cross-talk between tissues occurring in the regulation of metabolism in vivo. Although we measured the expression levels of genes corresponding to enzymes involved in the synthesis or degradation of endocannabinoids in adipose tissue, measurements in other tissues and plasma﻿ would also be valuable. That could further elucidate the overall activity of the peripheral endocannabinoid system including its autocrine, paracrine and endocrine functions [9, 49].

Furthermore, the collagenase isolation procedure might compromise the dexamethasone effects. However, the glucocorticoid receptor is located intracellularly and is not expected to be affected by collagenase acting in the extracellular environment. Still, it cannot be completely excluded that the dexamethasone effects are different between adipocytes isolated with collagenase and intact adipose tissue, respectively. Moreover, the lipolysis experiments involving dexamethasone and AM281 were performed only in female subjects, ruling out gender comparisons. However, as previously shown, dexamethasone treatment amplified adrenergic stimulation of lipolysis in adipocytes from women but not from men [50]. Female adipose tissue samples were therefore a priority in our experiments. Nonetheless, we plan to investigate males in future work on CNR1-mediated lipolysis regulation.

We selected the synthetic antagonist AM281 and the agonist ACEA because of their high affinity and specificity to the CNR1 receptor [51, 52]. However, other compounds with higher selectivity, e.g., SLV319 [53], could very well have more pronounced effects on lipolysis and glucose uptake than we have observed here.

Our data demonstrate that CNR1 and the endocannabinoid system in human adipose tissue is upregulated in states of insulin resistance, including T2D and glucocorticoid exposure. Also, our results suggest that CNR1 is involved in glucocorticoid-regulated lipolysis in subcutaneous adipocytes. This study gives further support to the concept of a role of the peripheral endocannabinoid system in insulin resistance, particulary in the context of high glucocorticoid exposure. The cannabinoid receptor type 1 in peripheral tissues may be an attractive drug target for the treatment of dyslipidemia and insulin resistance associated with T2D.

## Electronic supplementary material

Supplementary Information

Supplementary Figure

Supplementary Figure

Supplementary Figure

Supplementary Figure

Supplementary Figure

## Abbreviations

2-AG: 2-Arachidonoylglycerol
ACEA: Arachidonyl-2′-chloroethylamide
AM281: 1-(2,4-Dichlorophenyl)-5-(4-iodophenyl)-4-methyl-*N*-4-morpholinyl-1*H*-pyrazole-3-carboxamide
AEA: Anandamide
CNR1: Cannabinoid receptor type 1
CNR2: Cannabinoid receptor type 2
DAGL: Diacylglycerol lipase
ERK: Extracellular signal-regulated kinase
FAAH: Fatty acid amide hydrolase
HSL: Hormone-sensitive lipase
KRH: Krebs-Ringer media
MAPK: Mitogen-activated protein kinase
MGL: Monoacylglycerol lipase
NAPE-PLD: *N*-acyl phosphatidylethanolamine phospholipase D
OAT: Omental adipose tissue
SAT: Subcutaneous adipose tissue
T2D: Type 2 diabetes
