# Supplementary material for: Role of cannabinoid receptor 1 in human adipose tissue for lipolysis regulation and insulin resistance
Source: Endocrine. 2016 Nov 17;55(3):839–52. doi: 10.1007/s12020-016-1172-6 (PMC5316391; doi:10.1007/s12020-016-1172-6)
Supplement: Supplementary file 1 — Supplementary Information [file 12020_2016_1172_MOESM1_ESM.docx]

**Role of cannabinoid receptor 1 in human adipose tissue for lipolysis regulation and insulin resistance**

Journal: Endocrine

Cherno O Sidibeh^1^, Maria J Pereira^1^, Joey Lau Börjesson^1^, Prasad G Kamble^1^, Stanko Skrtic^2,3^, Petros Katsogiannos^1^, Magnus Sundbom^4^, Maria K Svensson^1^, Jan W Eriksson^1^

^1^Department of Medical Sciences, Uppsala University, Uppsala, Sweden

^2^AstraZeneca R&D, Mölndal, Sweden

^3^Dept of Endocrinology, Institute of Medicine, Sahlgrenska Academy, University of Gothenburg, Gothenburg, Sweden

^4^Department of Surgical Sciences, Uppsala University, Uppsala, Sweden

*Corresponding author contact info***:**

Jan W Eriksson

The Department of Medical Sciences, Uppsala University, 751 85 Uppsala, Sweden

Phone: +46 186114419

e-mail: [jan.eriksson@medsci.uu.se](mailto:jan.eriksson@medsci.uu.se)

**Supplementary Appendix**

**Supplementary Materials and Methods**

## Adipose tissue incubation

Moreover, SAT was incubated in DMEM (6 mM glucose, 10% FBS, 1% PEST) with or without the addition of dexamethasone (0.3 µM) for 24 h in 37°C, 5% CO_2_ and with or without the CNR1 antagonist/inverse agonist AM281 (1-(2,4-Dichlorophenyl)-5-(4-iodophenyl)-4-methyl-N-4-morpholinyl-1H-pyrazole-3-carboxamide, Sigma, 3 µM; K_i_=12 nM for CNR1 and 4200 nM for CNR2 [1]) for the final 4 h of incubation.

The adipose tissue was incubated with dexamethasone for 20 h prior to the addition of the CNR1 antagonist to induce insulin resistance and allow the CNR1 expression levels to be elevated before the antagonist was added. This is based on time curves generated in previous dexamethasone studies [2]. Addition of AM281 in the last 4 h of incubation was done to explore if inhibition of the CNR1 receptor could revert the glucocorticoid-induced insulin resistance in adipose tissue. SAT was also incubated with or without the CNR1 agonist ACEA (Arachidonyl-2'-chloroethylamide, Cayman, 1 µM; Ki=1.4 nM for CNR1 and Ki=2000 nM for CNR2 [3]) for 24 h.

## Lipolysis

Following incubation of adipose tissue adipocytes were isolated with collagenase (Sigma) and washed with Hank´s medium 199 (Gibco, Paisley, UK) supplemented with 4% BSA (Sigma), 150 nM adenosine (Sigma), pH 7.4. Adipocytes were diluted to a lipocrit of 2-3% and the cell suspension was incubated with or without isoproterenol (0.5 µM, Sigma) and with or without insulin (100 µU/mL, Actrapid, Novo Nordisk, Bagsvaerd, Denmark) in a gently shaking water-bath at 37 °C for 2 h. Glycerol released into the media was measured by colorimetric absorbance at 540 nm with Free Glycerol Reagent (Sigma). Cellular lipids were extracted [4] and adipocyte size and cell number was measured as previously described [5]. The glycerol released into the media was normalized per cell number and calculated relative to basal control in each experiment.

## Glucose uptake

After incubation of adipose tissue adipocytes were isolated with collagenase and washed in glucose-free Krebs-Ringer-Hepes (KRH), supplemented with 4% BSA, 150 nM adenosine, pH 7.4. Adipocytes were diluted ten times in KRH media and were stimulated or not with insulin (1000 µU/ml) for 15 min in a shaking water bath at 37 °C. Thereafter, D-[U-14C] glucose (0.26 mCi/L, 0.86 µM; NEC042V250UC, PerkinElmer, Waltham, Massachusetts, USA) was added and the accumulation of glucose followed for 45 min. The reaction was stopped by the transfer of the cell suspension into ice-cold tubes with silicon oil (SERVA Electrophoresis GmbH, Heidelberg, Germany) and the adipocyte-associated radioactivity was measured in a beta-counter. Glucose uptake was determined by the rate of transmembrane glucose transport and calculated as previously described [6].

## CNR1 gene and protein expression in 24 h incubated adipose tissue

Total RNA was isolated from adipose tissue using the RNeasy Lipid Tissue mini kit (Qiagen, Hilden, Germany). The RNA concentration was determined using the NanoDrop ND-1000 spectrophotometer (NanoDrop Technologies, Wilmington, DE, USA). RNA was converted to cDNA using high-capacity cDNA reverse transcription kit (Applied Biosystems, Foster City, CA, USA) and relative quantification of *CNR1* mRNA was performed. All reagents for *CNR1* real-time PCR (Assay-on-Demand: Hs01038522_s1), were purchased from Applied Biosystems and used according to the manufacturer's protocol. The relative quantification of *CNR1* gene expression was analyzed using the ABI Prism 7700HT Sequence Detection System (Applied Biosystems) or the CFX96 real-time PCR Detection System from Bio-Rad Laboratories (Hercules, CA, USA). A standard curve of pooled cDNA was used to determine the concentration of the target gene. The gene expression levels were normalized to the housekeeping gene 18S rRNA (Applied Biosystems).

For immunohistochemistry, adipose tissue that was incubated with or without dexamethasone for 24 h was snap frozen in liquid nitrogen. Frozen sections (12 µm) were mounted on glass slides (Superfrost^®^ Plus, Thermo Scientific, Braunschweig, Germany). Sections were fixated in ice-cold acetone:methanol (1:1) (acetone from Sigma, methanol from Merck Millipore, Billerica, MA, USA) for 10 min and washed in PBS-T (Medicago, Uppsala Sweden). The sections were blocked in a blocking solution containing phosphate buffered saline 1X (PBS; Medicago), 1% BSA (Sigma), 0.3 % Triton X-100 (Sigma), 0.1% Sodium Azide (Sigma) for 1 h followed by overnight incubation at 4 °C with the CNR1 primary antibody (Ab23703, Abcam, Cambridge, UK; diluted 1:300). Sections were washed in PBS-T (Medicago) and then allowed to incubate for 1 h at room temperature with secondary goat anti-rabbit Alexa fluor 594 (Life Technologies, Eugene, OR, USA; diluted 1:1000). Following incubation with the secondary antibody, the sections were mounted with coverslips by using ProLong Gold Antifade Mountant with DAPI (Life Technologies) and allowed to dry overnight in darkness. Sections were then visualized under a confocal microscope and images were captured with a Laser Scanning Microscope ZEISS LSM780 (Carl Zeiss AG, Oberkochen, Germany). The same adjustments to brightness and contrast were applied to all images.

**Supplementary Results**

## Associations between CNR1 gene expression in long-term incubated adipose tissue and metabolic parameters

In bivariate correlation analyses, *CNR1* gene expression in non-treated (control) SAT and OAT correlated positively with insulin, HOMA-IR, BMI and waist circumference and negatively with HDL-cholesterol (*p*<0.05, Table 2, Supplementary Fig. 2 and 3). In addition, *CNR1* gene expression in SAT correlated positively with subcutaneous and omental adipocyte diameter (Table 2). Significant variables in the bivariate correlations were included in multivariate regressions (Table 2). After adjustments in multivariate analyses, HOMA-IR (standard β coefficient=0.42, *p*<0.001), waist circumference (standard β coefficient=0.83) and omental adipocyte diameter (standard β coefficient=-0.45, *p*<0.001) remained significant predictors of *CNR1* gene expression in SAT (model: *r*^2^=0.81, *p*<0.001) (Table 2). In OAT only HOMA-IR (standard β coefficient=0.65, *p*<0.001) remained as a significant predictor of *CNR1* gene expression in omental adipose tissue (model: *r*^2^=0.43, *p*<0.001) (Table 2).

Also, dexamethasone-induced *CNR1* gene expression in SAT was found to positively correlate with insulin, HOMA-IR, BMI, waist circumference and subcutaneous and omental adipocyte diameter and negatively with HDL-cholesterol (*p<0.05*, Table 2 and Supplementary Fig. 2 and 3) in bivariate correlation analyses. Waist circumference (standard β coefficient=0.70, *p*<0.001, Table 2) remained the sole predictor in a multivariate stepwise regression (model: *r*^2^=0.49, *p*<0.001). Dexamethasone-induced *CNR1* gene expression in OAT did not correlate with any metabolic variable (Table 2).

Furthermore, *CNR1* gene expression was 2.8-fold higher in paired samples of non-treated SAT compared with OAT (control, *n*=41, *p*=0.003) and 1.2-fold higher in paired samples of dexamethasone-treated SAT compared with OAT (*n*=30, *p*=0.001) (Supplementary Fig. 2C). With the exception of a 2.5-fold higher dexamethasone-induced CNR1 expression in SAT from males compared with OAT from females (p=0.001, Supplementary Fig. 4), there were no significant differences in CNR1 expression levels between males and females or pre- and post-menopausal females across all BMI ranges.

**Supplementary Tables**

**Supplementary Table 1** - Clinical characteristics of study participants used for measurement of *CNR1* gene expression in freshly harvested adipose tissue (*n*=40).

|  | **Control** | | |  |  | | **Type 2 diabetes** | |  | **p** |
| --- | --- | --- | --- | --- | --- | --- | --- | --- | --- | --- |
|  | **Average** | | **Range** | | |  | **Average** | | **Range** |  |
| N | 20 (10 M, 10 F) | | | | |  | 20 (10 M, 10 F) | | | - |
| Age, years | 58 | 34-72 | | | |  | 58 | 41-71 | | 0.698 |
| BMI (Kg/m^2^) | 30.8 | 22.7-38.4 | | | |  | 30.7 | 22.5-39.9 | | 0.820 |
| Waist-hip-ratio | 0.96 | 0.84-1.09 | | | |  | 0.99 | 0.90-1.08 | | 0.201 |
| Sc adipocyte diameter (µm) | 109.6 | 91.4-124.7 | | | |  | 106.4 | 82.6-124.6 | | 0.512 |
| Plasma glucose (mmol/L) | **6.0** | **4.9-7.3** | | | |  | **8.2** | **6.1-11.5** | | **<0.001** |
| Serum insulin (mU/L) | **11.5** | **4.1-26.0** | | | |  | **15.5** | **4.1-31.0** | | **0.040** |
| HOMA-IR | **3.08** | **1.17-7.33** | | | |  | **5.27** | **1.25-10.83** | | **<0.01** |
| HbA_1c_, IFCC (mmol/mol) | **37.3** | **31-46** | | | |  | **48.8** | **37-73** | | **<0.001** |
| HbA_1c_ (%) | **5.6** | **5.0-6.4** | | | |  | **6.6** | **5.5-8.8** | | **<0.001** |
| P-total cholesterol (mmol/L) | 5.7 | 4.4-8.4 | | | |  | 4.9 | 3.1-6.8 | | 0.081 |
| P-HDL-cholesterol (mmol/L) | **1.3** | **0.9-1.9** | | | |  | **1.15** | **0.8-1.8** | | **0.046** |
| P-LDL-cholesterol (mmol/L) | 3.6 | 1.9-6.0 | | | |  | 3.1 | 1.8-4.9 | | 0.221 |
| P-triglycerides (mmol/L) | 1.6 | 0.7-3.5 | | | |  | 1.57 | 0.6-2.6 | | 0.947 |

Data are mean ± SD. Differences between groups were measured with MannWhitney U test.

Bold values indicate statistical significance (*p*<0.05).

Sc = subcutaneous; M= males; F= females.

Blood chemistry is fasting.

**Supplementary Table 2 –** Clinical characteristics of study participants whose adipose tissue explants were incubated and used for subsequent experiments (*n*=70).

| **Variable** | **Surgical biopsy^1^** | |  | **Needle biopsy^2^** | |
| --- | --- | --- | --- | --- | --- |
|  | **Mean** | **Range** |  | **Mean** | **Range** |
| Sex (male/female; n) | 13M/31F | - |  | 5M/21F | - |
| Age (years) | 47 | 24-66 |  | 53 | 21-72 |
| BMI (kg/m^2^) | 29.8 | 20.7-56.3 |  | 26.6 | 21.3-32.9 |
| Waist circumference (cm) | 100 | 71-180 |  | 91 | 77-115 |
| Subcutaneous adipocyte diameter (μm) | 98.5 | 63.8-127.8 |  | 109.8 | 96.1-126.6 |
| Omental adipocyte diameter (μm) | 88.5 | 50-130.9 |  | - | - |
| HbA_1c_ (mmol/mol, IFCC) | 34 | 24-43 |  | 35 | 27-43 |
| HbA_1c_ (%, NGSP) | 5.2 | 4.4-6.1 |  | 5.3 | 4.6-6.1 |
| Plasma glucose (mmol/L) | 5.1 | 3.8-6.8 |  | 5.9 | 5.2-7.7 |
| Serum insulin (mU/L) | 11.8 | 2.8-40 |  | 9.2 | 5.0-18.1 |
| HOMA-IR | 2.8 | 0.5-10.7 |  | 2.5 | 1.2-5.1 |
| Serum triglycerides (mmol/L) | 1.4 | 0.5-3.5 |  | 1.1 | 0.53-2.14 |
| Serum total cholesterol (mmol/L) | 5.2 | 2.6-7.6 |  | 5.6 | 3.8-8.2 |
| Serum LDL-cholesterol (mmol/L) | 3.2 | 1.1-5.3 |  | 3.4 | 2.0-5.8 |
| Serum HDL- cholesterol (mmol/L) | 1.4 | 0.6-3.2 |  | 1.5 | 1.0-2.2 |

^1^Paired samples of subcutaneous and omental adipose tissue used for long-term incubation with dexamethasone and measurement of *CNR1* gene expression and correlation analyses.

^2^Subcutaneous adipose tissue samples used for short- and long-term incubation with dexamethasone, AM281 and ACEA and measurements of lipolysis and glucose uptake.

HbA_1c_, glycosylated hemoglobin; HOMA-IR, homeostatic model assessment of insulin resistance index; HDL-cholesterol, high-density lipoprotein; LDL-cholesterol, low-density lipoprotein; BMI, body mass index.

**Supplementary Table 3 -** Clinical characteristics of study participants in lipolysis experiments (n=16).

|  | **Average** | **Range** |
| --- | --- | --- |
| Sex (n; male/female) | ^*^2M/16F |  |
| Age (years) | 49 | 21-72 |
| BMI (kg/m^2^) | 29.5 | 21.2-55.2 |
| Waist circumference (cm) | 96 | 77-135 |
| Subcutaneous adipocyte diameter (μm) | 110 | 96-126 |
| HbA_1c_ (mmol/mol, IFCC) | 33 | 27-41 |
| HbA_1c_ (%, NGSP) | 5.2 | 4.6-5.9 |
| Plasma glucose (mmol/L) | 5.7 | 5.2-6.6 |
| Serum insulin (mU/L) | 10.3 | 5.0-24.0 |
| HOMA-IR | 2.7 | 1.2-6.2 |
| Serum triglycerides (mmol/L) | 1.1 | 0.5-1.8 |
| Serum total cholesterol (mmol/L) | 5.1 | 3.7-7.8 |
| Serum LDL-cholesterol (mmol/L) | 3.1 | 2.0-5.1 |
| Serum HDL- cholesterol (mmol/L) | 1.5 | 0.8-2.1 |

HbA_1c_, glycosylated hemoglobin; HOMA-IR, homeostatic model assessment of insulin resistance index; HDL-cholesterol, high-density lipoprotein; LDL-cholesterol, low-density lipoprotein; BMI, body mass index.

** Adipose tissue was acquired by surgery in two of the subjects and by needle biopsies in the other subjects.*

**Supplementary Table 4 –** Long-term effects of dexamethasone and AM281 (20 ± 4 h) on adipocyte lipolysis.

| **^F^n** | ***Control**  **Basal** | **Dexa Basal** | **Dexa+AM281**  **Basal** | **Control**  **Iso** | **Dexa Iso** | **Dexa+AM281**  **Iso** | **Control**  **Iso+Ins** | **Dexa Iso+Ins** | **Dexa+AM281**  **Iso+Ins** |
| --- | --- | --- | --- | --- | --- | --- | --- | --- | --- |
| 1 | 1.00 | 0.99 | 0.90 | 4.96 | 10.62 | 8.90 | 3.74 | 4.79 | 3.98 |
| 2 | 1.00 | 1.22 | 1.27 | 3.03 | 6.58 | 5.37 | 1.56 | 2.97 | 2.48 |
| 3 | 1.00 | 1.31 | 1.33 | 3.11 | 2.91 | 3.00 | 2.09 | 2.78 | 2.25 |
| 4 | 1.00 | 0.95 | 0.37 | 2.80 | 3.92 | 2.88 | 2.30 | 2.81 | 2.90 |
| 5 | 1.00 | 0.79 | 0.75 | 8.01 | 8.54 | 6.16 | 5.88 | 5.81 | 4.19 |
| 6 | 1.00 | 1.03 | 1.03 | 2.81 | 4.82 | 4.47 | 1.99 | 3.16 | 2.25 |
| 7 | 1.00 | 1.02 | 1.76 | 27.33 | 32.57 | 24.74 | 19.98 | 21.88 | 16.98 |
| 8 | 1.00 | 0.66 | 0.87 | 11.16 | 15.23 | 11.79 | 7.50 | 7.21 | 7.50 |
| 9 | 1.00 | 1.05 | 2.08 | 16.04 | 24.29 | 16.92 | 11.67 | 12.61 | 11.16 |
| 10 | 1.00 | 1.43 | 1.57 | 4.02 | 8.82 | 8.47 | 2.57 | 5.17 | 4.70 |

Iso, Isoproterenol; Ins, Insulin; Dexa, Dexamethasone; AM281, (1-(2,4-Dichlorophenyl)-5-(4-iodophenyl)-4-methyl-N-4-morpholinyl-1H-pyrazole-3-carboxamide).

**^F^n -** *Only female subjects were included in this set of experiments.*

**All values relative to basal control which has an average absolute value of 45.52±34.58 nmol/10^5^ cells/h.*

**Supplementary Table 5 -** Acute effects (30 min) of AM281 on adipocyte lipolysis.

| **^F^n** | ***Control**  **Basal** | **AM281 Basal** | **Control**  **Iso** | **AM281 Iso** | **Control**  **Iso+Ins** | **AM281 Iso+Ins** |
| --- | --- | --- | --- | --- | --- | --- |
| 1 | 1.00 | 1.04 | 7.57 | 6.12 | 5.20 | 4.52 |
| 2 | 1.00 | 0.92 | 7.03 | 6.68 | 2.44 | 2.76 |
| 3 | 1.00 | 0.66 | 18.71 | 16.55 | 14.69 | 12.81 |
| 4 | 1.00 | 0.98 | 21.59 | 16.71 | 14.80 | 11.47 |
| 5 | 1.00 | 0.89 | 19.37 | 18.97 | 7.30 | 7.27 |

Iso, Isoproterenol; Ins, Insulin; AM281, 1-(2,4-Dichlorophenyl)-5-(4-iodophenyl)-4-methyl-N-4-morpholinyl-1H-pyrazole-3-carboxamide.

**^F^n -** *Only female subjects were included in this set of experiments.*

**All values relative to basal control which has an average absolute value of 47.09±37.66 nmol/10^5^ cells/h.*

**Supplementary Table 6 –** Long-term effects (24 h) of ACEA on adipocyte lipolysis.

| **n** | ***Control**  **Basal** | **ACEA Basal** | **Control**  **Iso** | **ACEA Iso** | **Control**  **Iso+Ins** | **ACEA Iso+Ins** |
| --- | --- | --- | --- | --- | --- | --- |
| ^F^1 | 1 | 1.28 | 2.81 | 3.96 | 1.99 | 3.06 |
| ^M^2 | 1 | 0.57 | 3.41 | 5.31 | 2.38 | 2.98 |
| ^F^3 | 1 | 1.44 | 4.55 | 5.38 | 2.06 | 2.49 |
| ^F^4 | 1 | 2.08 | 11.16 | 13.25 | 7.50 | 8.40 |
| ^F^5 | 1 | 1.42 | 16.04 | 15.98 | 11.67 | 13.64 |
| ^F^6 | 1 | 1.73 | 4.02 | 6.43 | 2.57 | 4.15 |
| ^M^7 | 1 | 0.59 | 15.71 | 17.57 | 7.50 | 9.72 |
| ^F^8 | 1 | 1.03 | 9.83 | 11.18 | 4.65 | 6.37 |

Iso, Isoproterenol; Ins, Insulin; ACEA, Arachidonyl-2'-chloroethylamide.

**^F^n -** *Female subject*

**^M^n -** *Male subject*

**All values relative to basal control which has an average absolute value of 52.60±36.10 nmol/10^5^ cells/h.*

**Supplementary Figures**

**
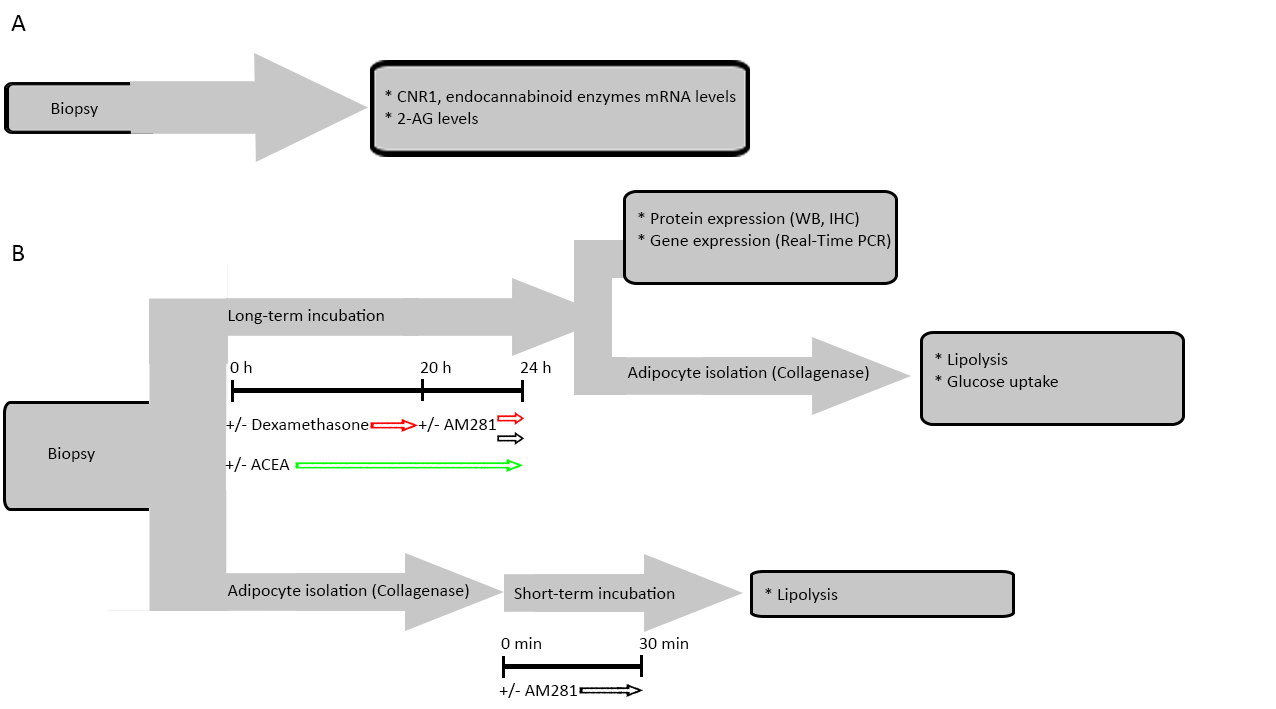
**

**Supplementary Figure 1 –** Simplified schematic figure of the general experimental design for short-term and long-term experiments (A) and for CNR1 mRNA expression levels in freshly harvested adipose tissue from type 2 diabetic and healthy controls **(B)**.

2-AG, 2 arachidonoylglycerol; WB, western blot; IHC, immunohistochemistry; AM281, 1-(2,4-Dichlorophenyl)-5-(4-iodophenyl)-4-methyl-N-4-morpholinyl-1H-pyrazole-3-carboxamide; ACEA, Arachidonyl-2'-chloroethylamide.


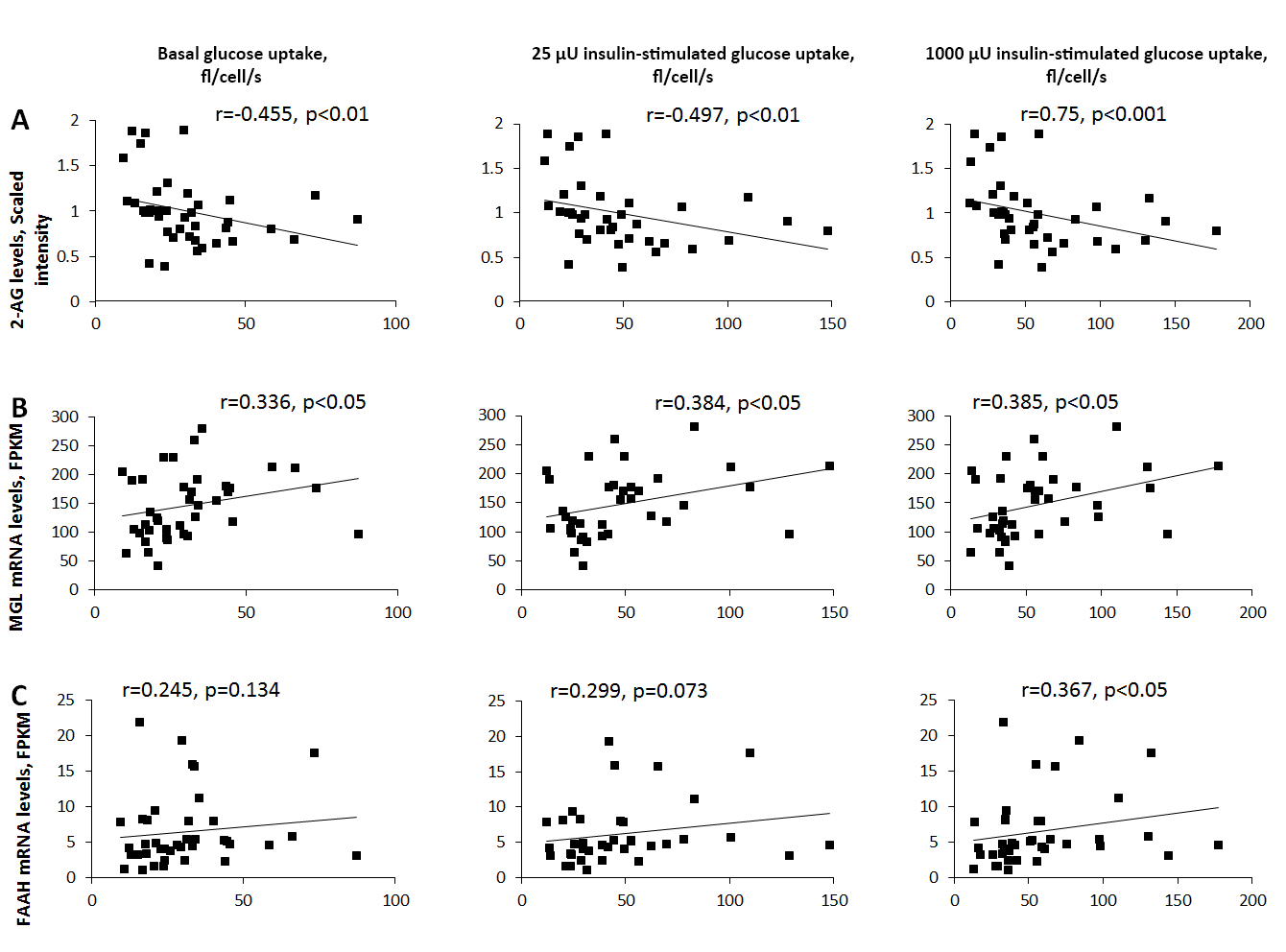


**Supplementary Figure 2 –** Bivariate correlation analyses between SAT levels of 2-AG (A) and gene expression levels of MGL (B) and FAAH (C) versus basal, 25 and 1000 µU insulin-stimulated glucose uptake in isolated adipocytes. SAT was freshly harvested.

2-AG, 2 arachidonoylglycerol; MGL, Monoacylglycerol lipase; FAAH, Fatty acid amide hydrolase


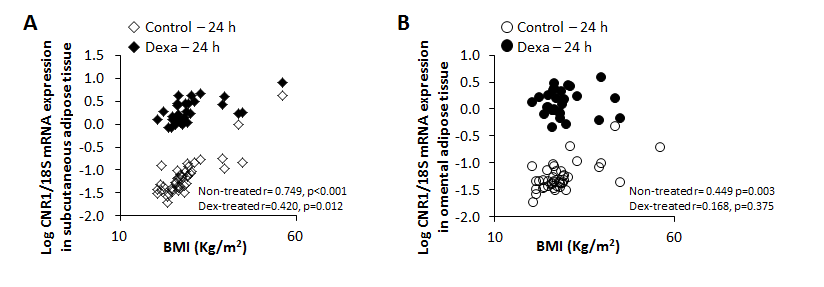


**Supplementary Figure 3 -** *CNR1* gene expression in 24 h incubated adipose tissue in relation to obesity. Linear regression between *CNR1* mRNA expression in non-treated (control, *n*=41) and dexamethasone-treated (*n*=30) for 24 h paired samples of subcutaneous **(A)** and omental adipose tissue **(B)** and BMI.


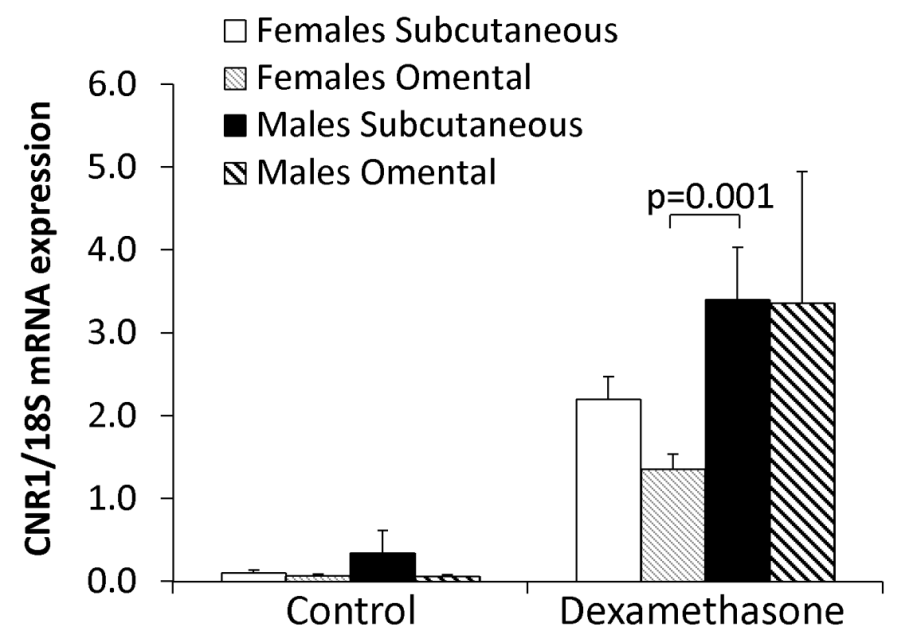


**Supplementary Figure 4 -** *CNR1* gene expression in subcutaneous and omental adipose tissue from females and males after 24 h incubation without (control) or with 0.3 µM dexamethasone. The data was log-transformed and analyzed with one-way analysis of variance with Tukey´s Multiple Comparison post-hoc test. Control: *n*=31 females and 15 males; Dexamethasone: *n*=24 females and 11 males.


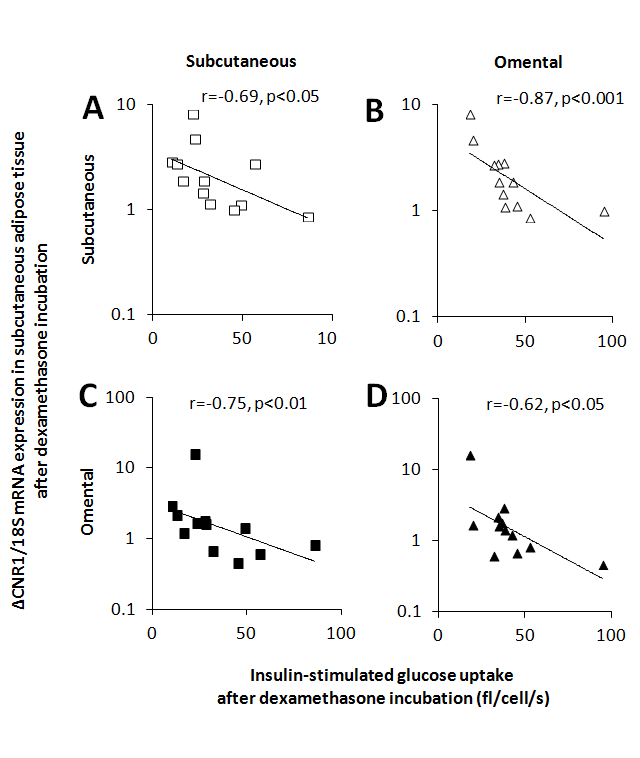


**Supplementary Figure 5 -** Correlations between the insulin-stimulated glucose uptake in dexamethasone-treated subcutaneous (**A** and **C**, squares) or omental (**B** and **D**, triangles) adipocytes versus the dexamethasone delta effect on subcutaneous (**A** and **B**, open symbols) or omental (**C** and **D**, filled symbols) *CNR1* gene expression in adipose tissue (*n*=12) paired from both depots.

**Supplementary References**

1. Lan, R., Gatley, J., Lu, Q., Fan, P., Fernando, S.R., Volkow, N.D., Pertwee, R., Makriyannis, A.: Design and synthesis of the CB1 selective cannabinoid antagonist AM281: a potential human SPECT ligand. AAPS pharmSci **1**(2), E4 (1999).

2. Lundgren, M., Buren, J., Ruge, T., Myrnas, T., Eriksson, J.W.: Glucocorticoids down-regulate glucose uptake capacity and insulin-signaling proteins in omental but not subcutaneous human adipocytes. The Journal of clinical endocrinology and metabolism **89**(6), 2989-2997 (2004). doi:10.1210/jc.2003-031157

3. Hillard, C.J., Manna, S., Greenberg, M.J., DiCamelli, R., Ross, R.A., Stevenson, L.A., Murphy, V., Pertwee, R.G., Campbell, W.B.: Synthesis and characterization of potent and selective agonists of the neuronal cannabinoid receptor (CB1). The Journal of pharmacology and experimental therapeutics **289**(3), 1427-1433 (1999).

4. Dole, V.P., Meinertz, H.: Microdetermination of long-chain fatty acids in plasma and tissues. The Journal of biological chemistry **235**, 2595-2599 (1960).

5. Lundgren, M., Svensson, M., Lindmark, S., Renstrom, F., Ruge, T., Eriksson, J.W.: Fat cell enlargement is an independent marker of insulin resistance and 'hyperleptinaemia'. Diabetologia **50**(3), 625-633 (2007). doi:10.1007/s00125-006-0572-1

6. Yu, Z.W., Jansson, P.A., Posner, B.I., Smith, U., Eriksson, J.W.: Peroxovanadate and insulin action in adipocytes from NIDDM patients. Evidence against a primary defect in tyrosine phosphorylation. Diabetologia **40**(10), 1197-1203 (1997). doi:10.1007/s001250050807

1. Pereira, M.J., Palming, J., Rizell, M., Aureliano, M., Carvalho, E., Svensson, M.K., Eriksson, J.W.: mTOR inhibition with rapamycin causes impaired insulin signalling and glucose uptake in human subcutaneous and omental adipocytes. Molecular and cellular endocrinology **355**(1), 96-105 (2012). doi:10.1016/j.mce.2012.01.024

2. Orr, J.S., Kennedy, A.J., Hasty, A.H.: Isolation of adipose tissue immune cells. Journal of visualized experiments : JoVE(75), e50707 (2013). doi:10.3791/50707

3. Nakada, M.T., Stadel, J.M., Poksay, K.S., Crooke, S.T.: Glucocorticoid regulation of beta-adrenergic receptors in 3T3-L1 preadipocytes. Molecular pharmacology **31**(4), 377-384 (1987).

4. Widmer, I.E., Puder, J.J., Konig, C., Pargger, H., Zerkowski, H.R., Girard, J., Muller, B.: Cortisol response in relation to the severity of stress and illness. The Journal of clinical endocrinology and metabolism **90**(8), 4579-4586 (2005). doi:10.1210/jc.2005-0354

5. Lan, R., Gatley, J., Lu, Q., Fan, P., Fernando, S.R., Volkow, N.D., Pertwee, R., Makriyannis, A.: Design and synthesis of the CB1 selective cannabinoid antagonist AM281: a potential human SPECT ligand. AAPS pharmSci **1**(2), E4 (1999).

6. Lundgren, M., Buren, J., Ruge, T., Myrnas, T., Eriksson, J.W.: Glucocorticoids down-regulate glucose uptake capacity and insulin-signaling proteins in omental but not subcutaneous human adipocytes. The Journal of clinical endocrinology and metabolism **89**(6), 2989-2997 (2004). doi:10.1210/jc.2003-031157

7. Hillard, C.J., Manna, S., Greenberg, M.J., DiCamelli, R., Ross, R.A., Stevenson, L.A., Murphy, V., Pertwee, R.G., Campbell, W.B.: Synthesis and characterization of potent and selective agonists of the neuronal cannabinoid receptor (CB1). The Journal of pharmacology and experimental therapeutics **289**(3), 1427-1433 (1999).

8. Dole, V.P., Meinertz, H.: Microdetermination of long-chain fatty acids in plasma and tissues. The Journal of biological chemistry **235**, 2595-2599 (1960).

9. Lundgren, M., Svensson, M., Lindmark, S., Renstrom, F., Ruge, T., Eriksson, J.W.: Fat cell enlargement is an independent marker of insulin resistance and 'hyperleptinaemia'. Diabetologia **50**(3), 625-633 (2007). doi:10.1007/s00125-006-0572-1

10. Yu, Z.W., Jansson, P.A., Posner, B.I., Smith, U., Eriksson, J.W.: Peroxovanadate and insulin action in adipocytes from NIDDM patients. Evidence against a primary defect in tyrosine phosphorylation. Diabetologia **40**(10), 1197-1203 (1997). doi:10.1007/s001250050807
