# Supplementary figures and images for: Role of cannabinoid receptor 1 in human adipose tissue for lipolysis regulation and insulin resistance
Source: Endocrine. 2016 Nov 17;55(3):839–52. doi: 10.1007/s12020-016-1172-6 (PMC5316391; doi:10.1007/s12020-016-1172-6)

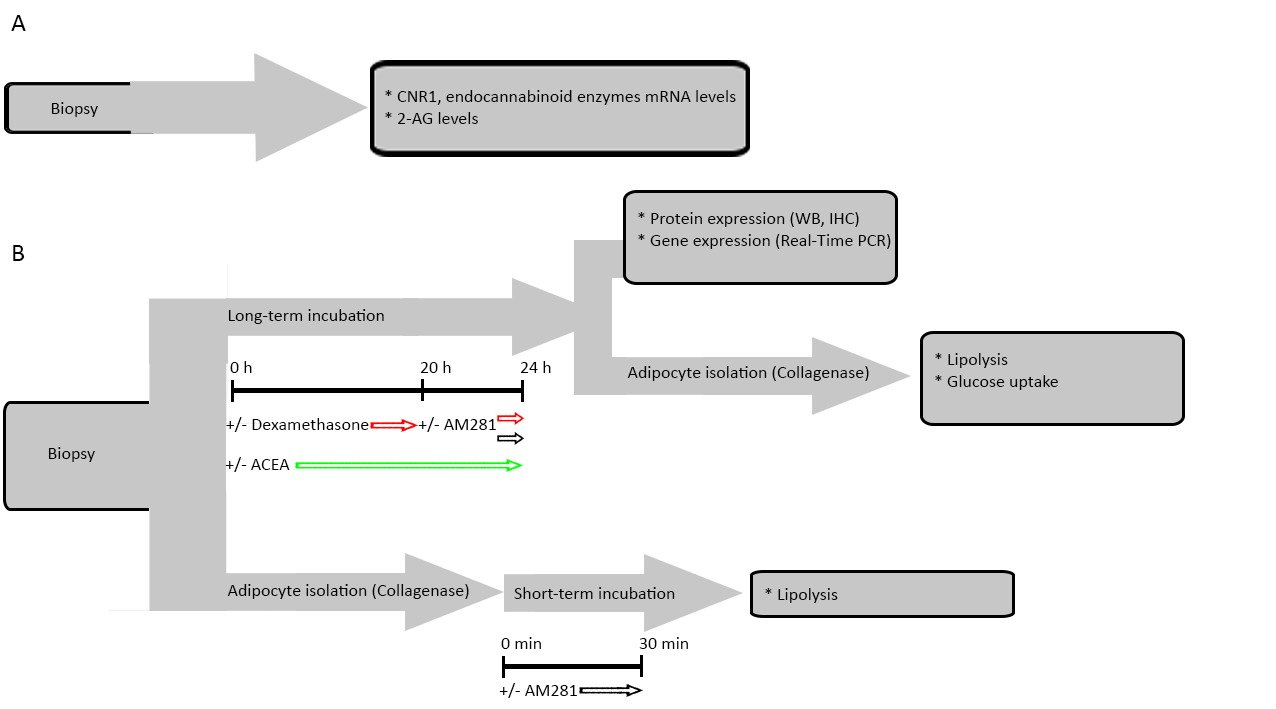

Supplement: Supplementary file 2 — Supplementary Figure [file 12020_2016_1172_MOESM2_ESM.tif]

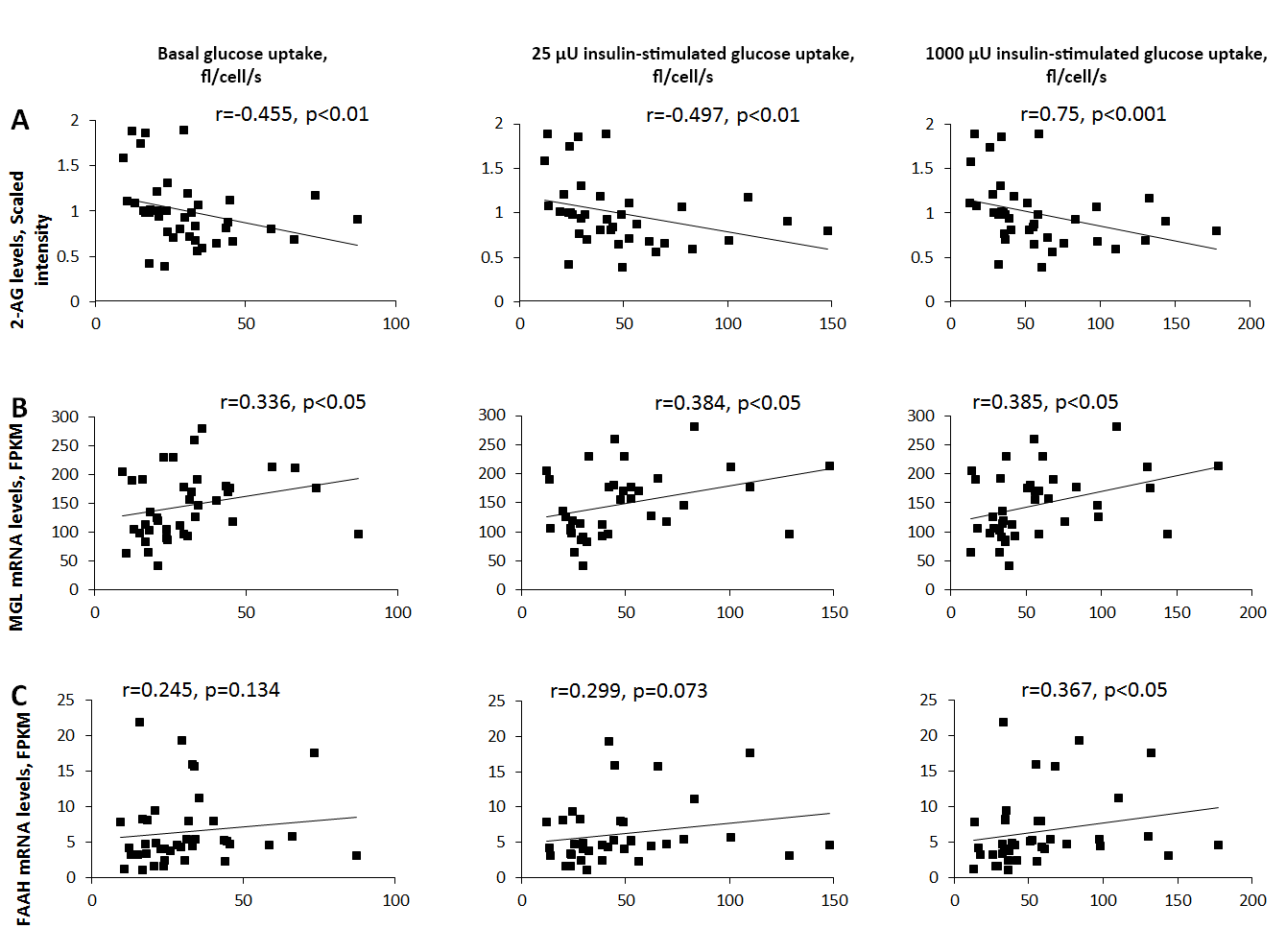

Supplement: Supplementary file 3 — Supplementary Figure [file 12020_2016_1172_MOESM3_ESM.tif]

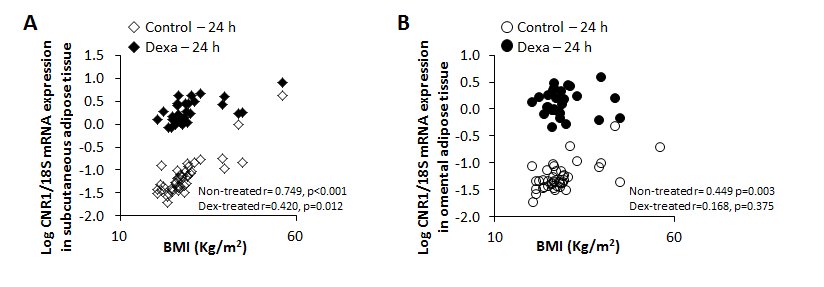

Supplement: Supplementary file 4 — Supplementary Figure [file 12020_2016_1172_MOESM4_ESM.tif]

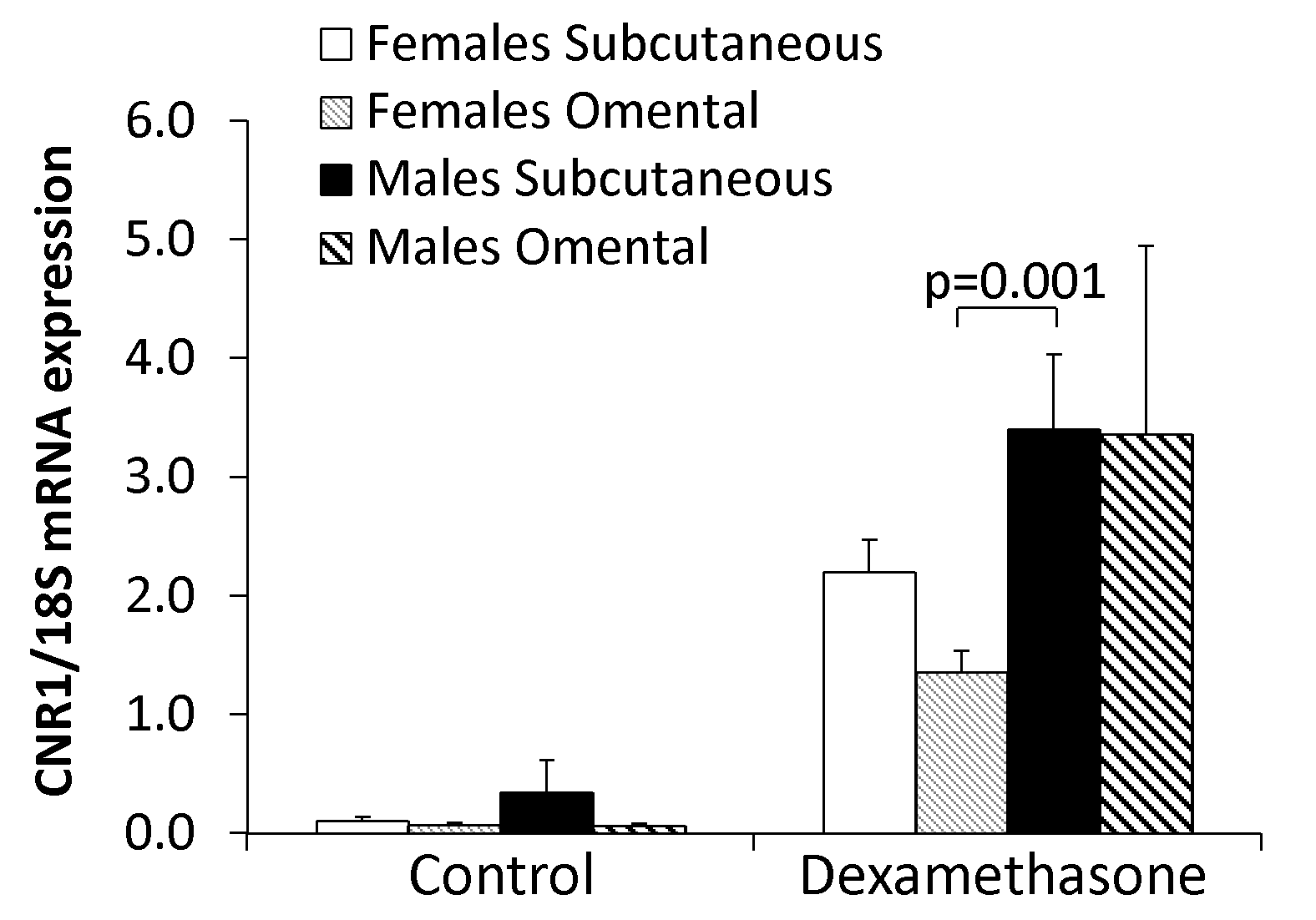

Supplement: Supplementary file 5 — Supplementary Figure [file 12020_2016_1172_MOESM5_ESM.tif]

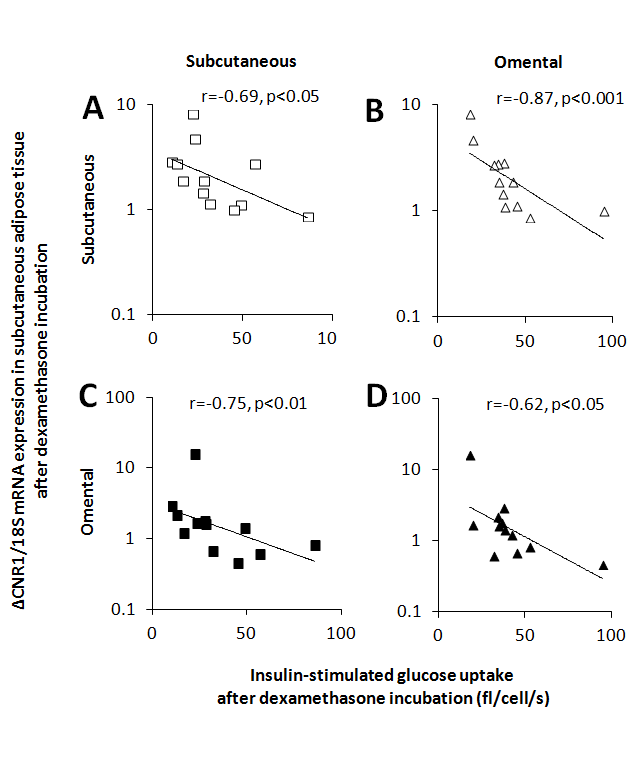

Supplement: Supplementary file 6 — Supplementary Figure [file 12020_2016_1172_MOESM6_ESM.tif]
